# Supplementary material for: Microarray-Based Prediction of Polycythemia after Exposure to High Altitudes
Source: Genes (Basel). 2022 Jul 2;13(7):1193. doi: 10.3390/genes13071193 (PMC9316656; doi:10.3390/genes13071193)
Supplement: Supplementary file 1 [file genes-13-01193-s001.zip › genes-1752435-supplementary.pdf]

Supplement Table S1. The log2(FC) of LRRC18 and HCAR3 in different compared group.

	D03 vs D0	D07 vs D0	D30 vs D0
LRRC18	−0.471467	−1.176533	−0.770763
HCAR3	1.477756	1.104452	0.129046

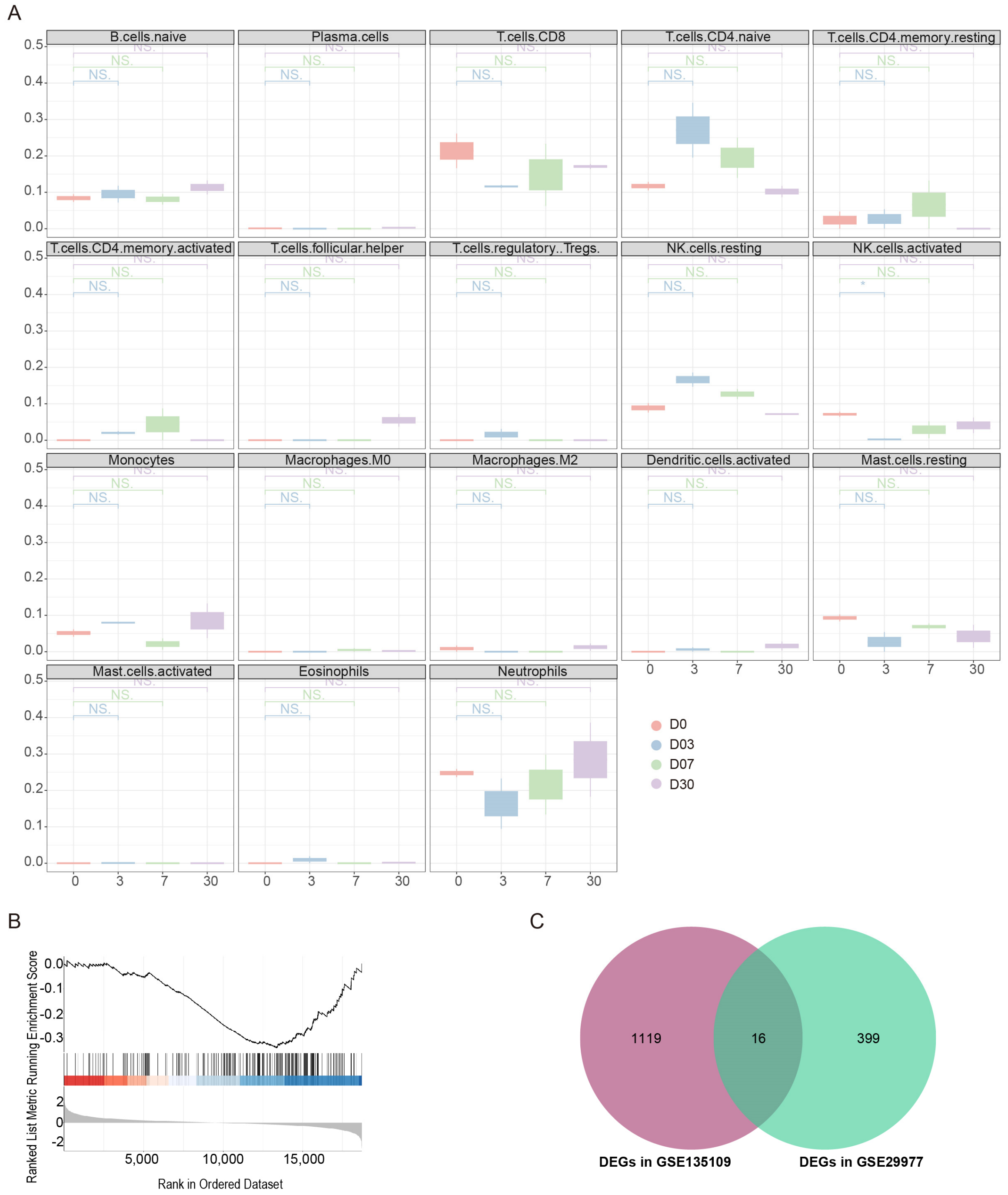

Figure S1: (**A**) The relative proporion of immune cell types in each group; (**B**) GSEA plot of geneset of "HALLMARK HEME METABOLISM"; (**C**) VENN plot of DEGs in two datasets.

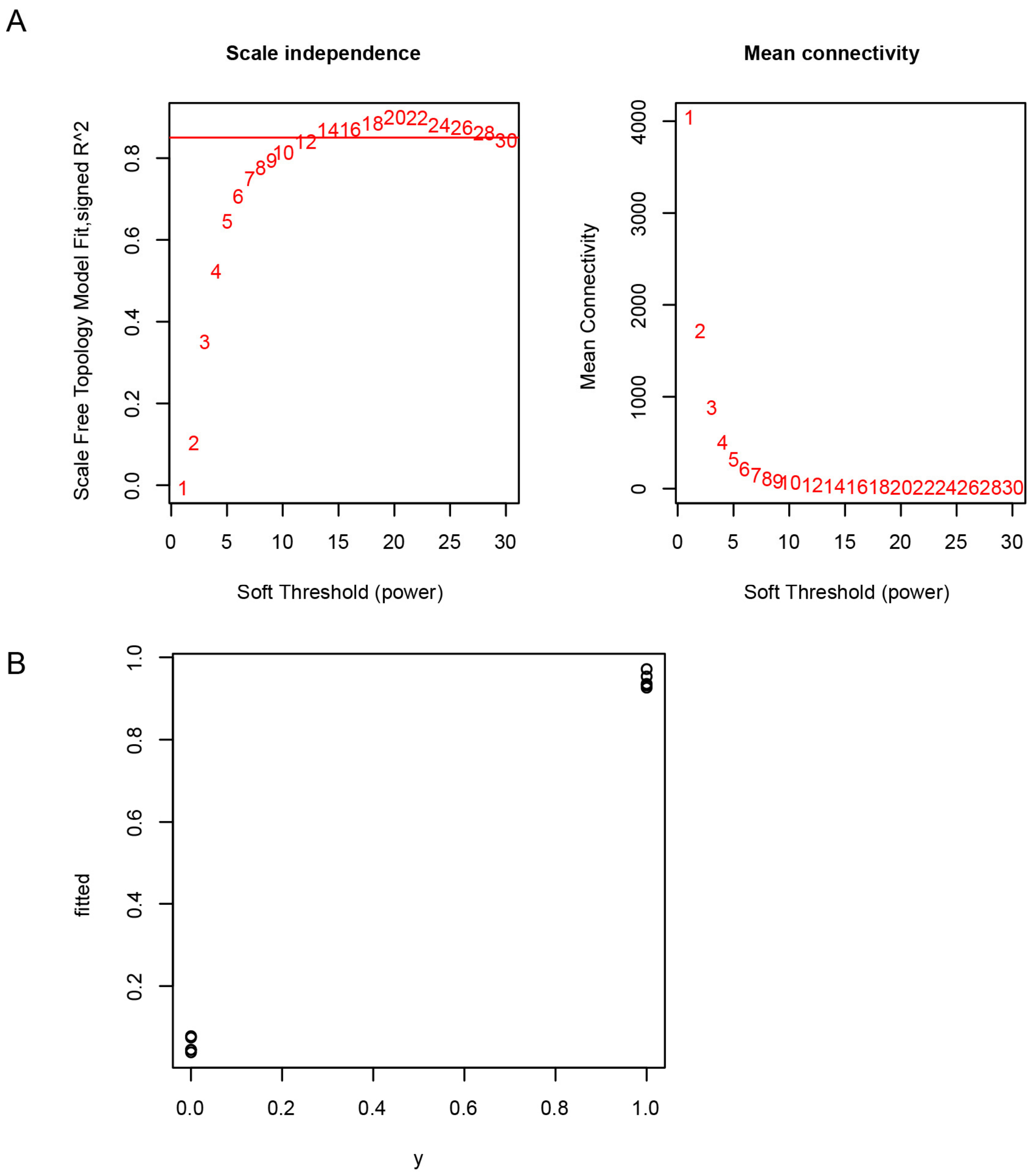

Figure S2: (**A**) Determination of soft-thresholding power in the weighted gene co-expression network analysis (WGCNA) (**B**) The result of cross-validate prediction. y indicates real disease state, fitted indicates the predict result of Logistic model.
